# Spatiotemporal Variations of the Frequency–Magnitude Distribution in the 2019 *M*_w_ 7.1 Ridgecrest, California, Earthquake Sequence

**DOI:** 10.3390/e25121612

**Published:** 2023-12-01

**Authors:** Eirini Sardeli, Georgios Michas, Kyriaki Pavlou, Filippos Vallianatos

**Affiliations:** 1 Section of Geophysics-Geothermics, Department of Geology and Geoenvironment, National and Kapodistrian University of Athens, 15772 Athens, Greece; eirsard@geol.uoa.gr (E.S.); kpavlou@geol.uoa.gr (K.P.); fvallian@geol.uoa.gr (F.V.); 2 Institute of Physics of Earth’s Interior and Geohazards, UNESCO Chair on Solid Earth Physics and Geohazards Risk Reduction, Hellenic Mediterranean University Research & Innovation Center, 73133 Chania, Greece

**Keywords:** Ridgecrest, earthquake sequence, frequency–magnitude distribution, fragment–asperity model, Tsallis entropy, non-extensive statistical physics, complexity

## Abstract

Significant seismic activity has been witnessed in the area of Ridgecrest (Southern California) over the past 40 years, with the largest being the *M*_w_ 5.8 event on 20 September 1995. In July 2019, a strong earthquake of *M*_w_ 7.1, preceded by a *M*_w_ 6.4 foreshock, impacted Ridgecrest. The mainshock triggered thousands of aftershocks that were thoroughly documented along the activated faults. In this study, we analyzed the spatiotemporal variations of the frequency–magnitude distribution in the area of Ridgecrest using the fragment–asperity model derived within the framework of non-extensive statistical physics (NESP), which is well-suited for investigating complex dynamic systems with scale-invariant properties, multi-fractality, and long-range interactions. Analysis was performed for the entire duration, as well as within various time windows during 1981–2022, in order to estimate the *q_M_* parameter and to investigate how these variations are related to the dynamic evolution of seismic activity. In addition, we analyzed the spatiotemporal *q_M_* value distributions along the activated fault zone during 1981–2019 and during each month after the occurrence of the *M*_w_ 7.1 Ridgecrest earthquake. The results indicate a significant increase in the *q_M_* parameter when large-magnitude earthquakes occur, suggesting the system’s transition in an out-of-equilibrium phase and its preparation for seismic energy release.

## 1. Introduction

**Figure 1:**
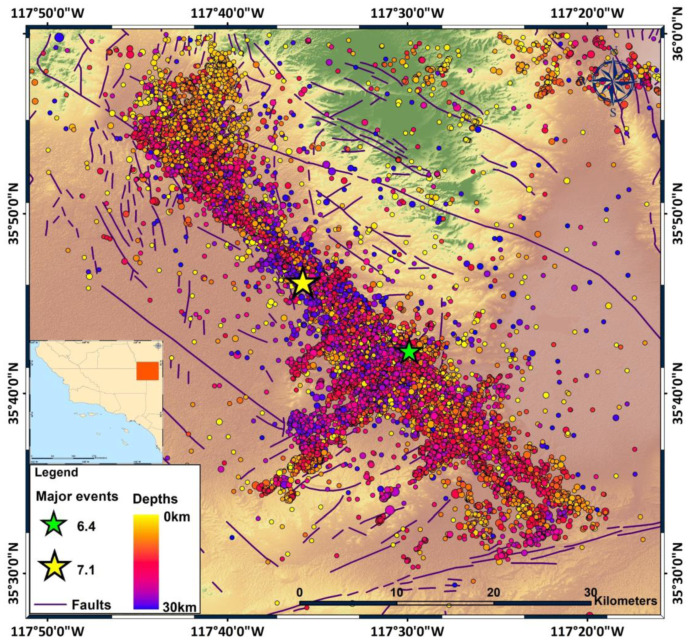
The spatial distribution of the 2019 Ridgecrest earthquake sequence for 38,452 events that occurred during the period between 4 July 2019 and 31 December 2019. The green and yellow stars indicate the foreshock and mainshock of magnitudes 6.4 and 7.1, respectively. Regional faults are marked with solid purple lines (https://koordinates.com/layer/701-california-faults/, accessed on 26 June 2023), and the seismic events are colored according to depth.

The 2019 Ridgecrest earthquake sequence took place in the eastern California shear zone, near the town of Ridgecrest and southwest of Searles Valley. The sequence initially evolved as a series of foreshocks, with the largest one of magnitude *M*_w_ 6.4 on 4 July 2019, preceding a strong mainshock of magnitude *M*_w_ 7.1 that occurred two days later, on 6 July 2019 (3:19:53 UTC). The *M*_w_ 7.1 event was accompanied by thousands of aftershocks during the following months (Figure 1) [1,2,3]. The spatial distribution of the two events of strong magnitudes, *M*_w_ 6.4 and *M*_w_ 7.1, as well as the thousands of aftershocks, revealed the activation of two main strike-slip fault zones: a previously unnoticed NE–SW left-lateral strike-slip fault zone associated with the *M*_w_ 6.4 seismic event and a NW–SE right-lateral strike-slip Little Lake fault zone associated with the *M*_w_ 7.1 mainshock [1,4,5,6]. The activated area is situated near the Airport Lake and Little Lake fault zones, both of which have a lengthy history of seismic activity [7]. More specifically, the activated fault zone displays widespread orthogonal faulting over multiple length scales, characterized by intricate geometric patterns [1]. The largest scale is approximately 55 km in a northwest-striking direction, intersected orthogonally by a fault roughly 15 km in length [1,4]. During the *M*_w_ 7.1 earthquake, the larger of these structures was the main one that ruptured, whereas, the *M*_w_ 6.4 event affected the smaller structure [1,4].

The rupture processes of the *M*_w_ 6.4 and the *M*_w_ 7.1 strong events have also been studied using geodetic and seismic data, revealing a complex interaction between multiple fault segments and branches, as well as the spatial and temporal variations of slip, stress drop, rupture speed, and directivity [2,4,5,8]. The 2019 Ridgecrest earthquake sequence has provided an exceptional occasion to investigate the physics of faulting and earthquake interactions in a complex fault system.

Over the past forty years, seismic activity in the Ridgecrest area has been characterized by swarms of earthquakes, with some lasting for over a year, and some notable moderate-magnitude events, such as the 1982 *M*_L_ 4.9 Indian Wells Valley event and the 1995–1996 Ridgecrest sequence, including three *M*_w_ 5+ earthquakes [9]. The 1995 earthquake sequence began on 17 August 1995, with an earthquake of magnitude *M*_w_ 5.4, followed by a *M*_w_ 5.8 event on 20 September 1995 and a *M*_w_ 5.2 event on 7 January 1996. The 1995 events occurred on normal and strike-slip NW- and NE-trending faults [9], showing similar complexity to the 2019 events.

In the present study, Ridgecrest’s seismicity is being investigated in terms of the frequency–magnitude distribution (FMD), which is an inherent component of the regional level of seismic activity and a fundamental part of seismic hazard assessments. The analysis of the FMD is performed with the fragment–asperity (F–A) model, initially developed by Sotolongo-Costa and Posadas [10] within the framework of non-extensive statistical physics (NESP) [11]. Using the F–A model, we analyzed the spatiotemporal variations of the derived entropic index *q_M_*, which is used as an index for the physical state of the studied region. The F–A model is used to calculate the seismic energy distribution function (EDF) utilizing fragment size distribution, providing an EDF that includes the Gutenberg–Richter (G–R) scaling relation as a specific case [12,13].

The *q_M_* parameter is herein estimated for the entire duration and also for various time windows, during the period 1981–2022. The variations of *q_M_* are examined to identify patterns associated with the evolution of the seismic activity and the results are subject to discussion. Additionally, we estimated the spatiotemporal variations in *q_M_* values derived from the F–A model for the Ridgecrest earthquake sequence. We analyzed the background seismicity from 1981 to 2019 (before the foreshock of *M*_w_ 6.4 on 4 July 2019) to estimate the background *q_M_* values during this period, and then for the period after the foreshock of *M*_w_ 6.4, before the occurrence of the *M*_w_ 7.1 mainshock, and finally, after the *M*_w_ 7.1 mainshock on 6 July 2019 to November 2019 for each month of aftershock activity.

## 2. Methodological Approach Based on NESP

### Non-Extensive Fragment–Asperity Model for Seismic Energies

In 2004, Sotolongo-Costa and Posadas [10] introduced the fragment–asperity (F–A) model of earthquake interactions, outlining the earthquake generation mechanism based on the small-scale processes within fault zones. This general model [10], developed within the non-extensive statistical physics (NESP) framework, considers the interaction between two rough profiles (fault blocks) and the fragments that occupy the space between them, caused by the local fracturing of tectonic plates. The fragments can have a significant impact on the earthquake triggering process. As stress between two fault surfaces rises, these rough fragments act both as roller bearings, expediting the slipping process, whereas when a fragment is displaced or an asperity brakes, the fault planes slip and seismic energy releases. Since fragments result from the violent fractioning between fault planes, it is anticipated that there will be long-range interactions between all parts of existent fragments. This implies that the size distribution function of the fragments is more appropriately treated using the NESP formalism.

NESP, introduced by Tsallis [14,15,16], is proposed as a possible generalization of Boltzmann–Gibbs (BG) statistical physics and has found wide applications in various non-linear dynamical systems [11]. Furthermore, the non-additive Tsallis entropy *Sq,* includes the parameter *q*, which quantifies the non-extensivity of a system. In the limit, where *q* = 1, *Sq* approaches the BG entropy. Even though *Sq* and *S*_BG_ have several common characteristics, such as non-negativity, expansibility, and concavity, there is a characteristic dissimilarity between the two entropies. The BG entropy is additive, signifying that the entropy of a combined system is the sum of the entropies of its individual parts, whereas the Tsallis entropy *Sq* is non-additive. In addition, the equilibrium phase of various short-range interacting systems (e.g., Hamiltonians) is well approximated with BG statistical physics, whereas various non-linear long-range interacting systems are better described with NESP [16,17].

Following Sotolongo-Costa and Posadas [10], the non-additive entropy *Sq*, in terms of the probability *p*(*σ*) of finding a fragment of area *σ*, is presented as follows:

(1) $$S_{q}=k\frac{1-\int p^{q}\left(\sigma \right)d\sigma }{q-1}$$

where *q* is the non-extensive parameter. Silva et al. [18] introduced the fragment size distribution function as:

(2) $$p\left(\sigma \right)={\left[1-\frac{\left(1-q\right)}{\left(2-q\right)}(\sigma -\sigma_{q})\right]}^{\frac{1}{1-q}}$$

Moreover, Silva et al. [18] introduced a scaling law between the released relative energy *Ε* and the volume of fragments *r* with the relationship *E*~*r*^3^, in agreement with the standard seismic moment theory [19]. The proportionality between the released energy *E* and *r*^3^ can then be expressed as:

(3) $$\sigma -\sigma_{q}={\left(\frac{Ε}{A}\right)}^{\frac{2}{3}}$$

where *σ* scales with *r*^2^, and *A* is proportional to the volumetric energy density.

Using Equations (2) and (3), the energy distribution function (EDF) of earthquakes is derived as:

(4) pE=C1Ε131+C2Ε231q−1

with C1=23A23 and C2=−(1−q)(2−q)A23

Telesca [20] further used the relation M=2/3log⁡Ε to derive the cumulative magnitude distribution:

(5) $$log\left(\frac{N(>M)}{N}\right)=\frac{2-q_{M}}{1-q_{M}}log\left[1-\left(\frac{1-q_{M}}{2-q_{M}}\right)\left(\frac{10^{M}}{A^{2/3}}\right)\right]$$

Furthermore, by considering the threshold magnitude (*Mc*), Telesca [13] introduced a modified function that links the cumulative number of earthquakes with magnitude, expressed as:

(6) $$log\left(\frac{N(>M)}{N}\right)=\frac{2-q_{M}}{1-q_{M}}log\left(\frac{1-\left(\frac{1-q_{M}}{2-q_{M}}\right)\left(\frac{10^{M}}{A^{2/3}}\right)}{1-\left(\frac{1-q_{M}}{2-q_{M}}\right)\left(\frac{10^{Mc}}{A^{2/3}}\right)}\right)$$

Temporal variations and an increase in *q_M_* suggest that the seismic area’s physical state is departing from equilibrium [21,22,23]. The fitting procedure of Equation (6) to the observed distribution, is the Levenberg–Marquardt non-linear least-square method [24,25] which is used to solve non-linear least squares problems. As mentioned in [26], this approach is widely known for its effectiveness in achieving high precision and swiftly converging to the best possible solution. The F–A model has found utility in diverse applications, including regional and local seismic activity, as well as volcanic seismicity [21,22,27,28].

According to Telesca [13], the maximum likelihood *q_M_* values are linked to the maximum likelihood Gutenberg–Richter *b* value, as follows:

(7) $$b=\frac{2-q_{M}}{q_{M}-1}$$

which is equivalent to the relation derived by [23].

As it is commonly known, the computation of *b* value using the maximum likelihood approach [29] is highly sensitive to the initial choice of minimum earthquake magnitude *Mc* in the seismic catalogue. On the other hand, *q_M_* value estimation is relatively stable irrespective of the choice of *Mc* [30].

## 3. Seismological Data

In this work, we used the waveform relocation catalogue for Southern California [31] provided by the Southern California Earthquake Data Center (SCEDC), which expands from 1981 to 2022. A total of 103,706 earthquakes, which occurred in the period between 3 January 1981 and 31 March 2022, within a defined boundary of 117.2° E~118.0° E longitude and 35.4° N~36.0° N latitude, were considered. The depth distribution of seismicity varies from –1.41 to 30.8 km, whereas the magnitudes range between –1.02 and 7.1.

The research area has experienced thousands of small events, with some of the largest in the magnitude range of 4 to 7. On 4 July 2019, a *M*_w_ 6.4 earthquake occurred, preceded by a series of small events in the prior hour. The largest earthquake occurred 34 h later, on 6 June, with a magnitude of *M*_w_ 7.1. Eleven months later, on 4 June 2020, a *M*_w_ 5.53 aftershock took place to the east of Ridgecrest (Figure 1). Over the past 40 years, 11 other *M* > 5 earthquakes have occurred in this area. The largest one was an earthquake of magnitude *M*_w_ 5.8 on 20 September 1995, about 13 km to the west–northwest of Ridgecrest [31]. In addition, according to the catalogue, an earthquake of magnitude *M*_w_ 5.2 occurred on 1 October 1982.

**Figure 2:**
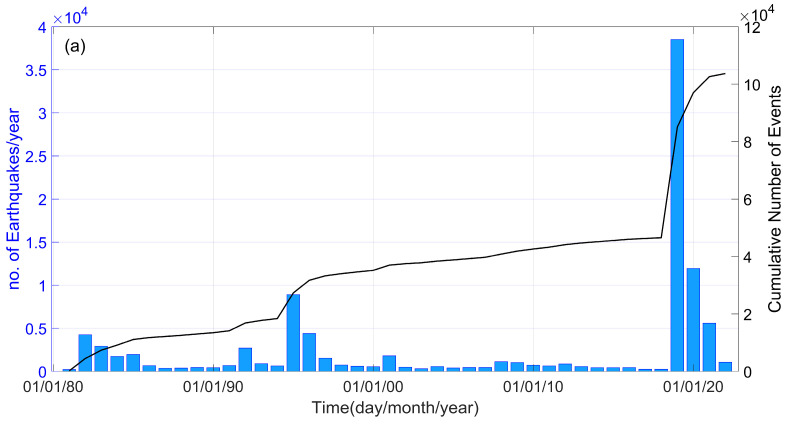

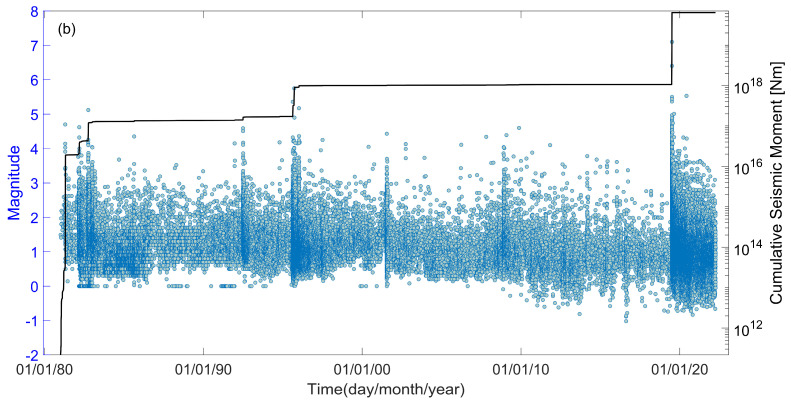
(**a**) Histogram showing the annual number of events during the period between January 1981 and April 2022. The black line illustrates the cumulative number of seismic events, N, in the research area. (**b**) Magnitude distribution per day versus time. The black line shows the cumulative seismic moment release.

Within the study area, there were a total of 8924 events in 1995, with 2722 of them occurring in September of that year (Figure 2a). In 2019, there was a seismic burst of 38,530 events associated with the Ridgecrest earthquake sequence, with 6 of them over a magnitude of *M* 5. Figure 2b illustrates the temporal progression of the earthquake magnitudes in the Ridgecrest area, as well as the cumulative seismic moment release throughout the 41-year observation period. The equation logMo=1.5×M+9.1 was used to approximate the seismic moment release (in Nm) in relation to earthquake magnitudes [32].

## 4. Results

### 4.1. Frequency–Magnitude Distribution during 1981–2022

**Figure 3:**
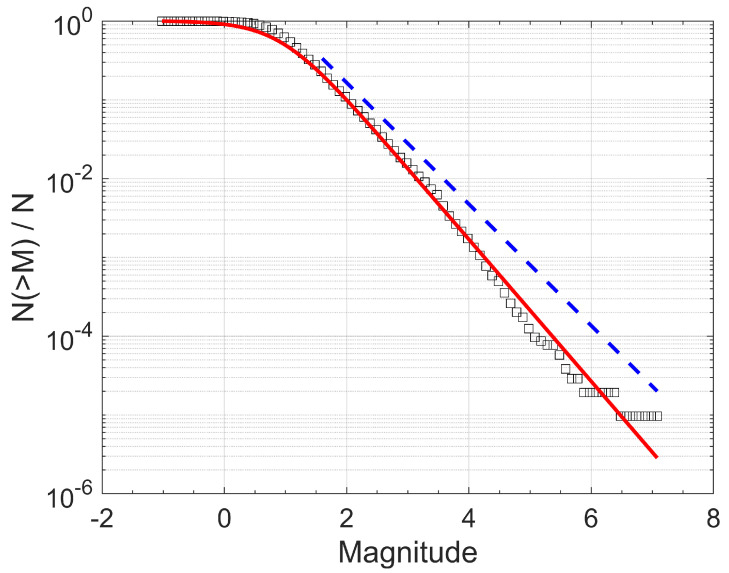
Normalized cumulative magnitude distribution (squares) of the 1981–2022 earthquake activity in Ridgecrest and the model of Equation (6) (red solid line) for the values of *q_M_* = 1.52 ± 0.01 and *A* = 27.92 ± 8.04. The blue dashed line illustrates the Gutenberg–Richter relation for *b* = 0.771 ± 0.005 and *α* = 5.561 ± 0.077.

The F–A model, as discussed in the preceding section, is applied to the normalized cumulative magnitude distribution for our dataset from 1981 to 2022, for the entire magnitude range. This model describes quite well the observed magnitude distribution, while fitting Equation (6) to the observed data provides the values *q_M_* and *A* using a non-linear squares algorithm. The results of this analysis are presented in Figure 3. The F–A model fits well the data for the values of *q_M_* = 1.52 ± 0.01 and *A* = 27.92 ± 8.04.

One of the most renowned empirical scaling relations in seismology is the Gutenberg–Richter (G-R) [33] relation, which expresses a power-law dependence between the number of earthquakes *N* and the released seismic energies *E*. In terms of earthquake magnitudes *M*, it is expressed as $N\left(>M\right)=10^{a-bM}$, where *N*(>*M*) is the number of earthquakes greater than a threshold magnitude *M*, and *a*, *b* are positive scaling parameters. The *b* value is usually calculated using the maximum likelihood method [29], as subsequently amended by [34], as: $b=(1/\bar{M}-M_{0})\log e$, where $\bar{M}$ is the observed mean magnitude and *M*_0_ is the minimum magnitude. Therefore, for comparison, a maximum likelihood fit that corresponds to the G–R relation is also plotted in Figure 3, for the values of *M*_0_ = 1.6, *b* = 0.771 ± 0.005, and *α* = 5.561 ± 0.077.

### 4.2. Variations of q_M_ Values with Time

**Figure 4:**
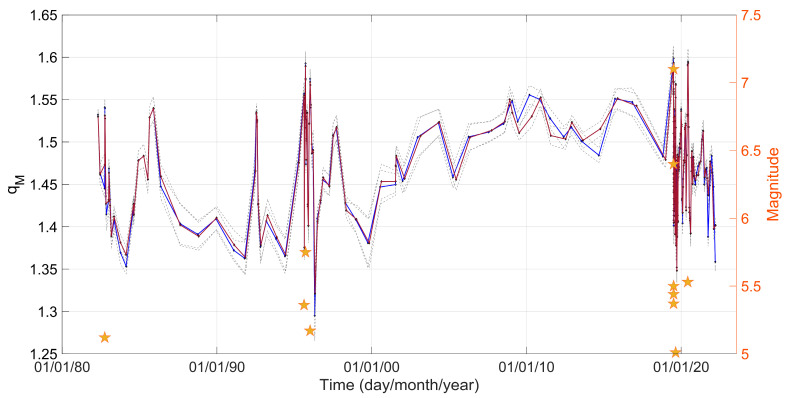
Temporal evolution of the *q_M_* value for all the seismic events (blue solid line) and for *M* ≥ *Mc* (red solid line), along with their corresponding standard deviations (gray dashed lines), calculated in successive time intervals with a 50% overlap covering the period from 1981 to 2022. Stars indicate earthquakes of a magnitude greater than 5.

The F–A model is initially applied to the seismic catalogue for the entire period from 1981 to 2022. A notable aspect in this analysis is to examine the temporal variations of the *q_M_* parameter and how these variations are related to the dynamic evolution of earthquake activity, which can offer valuable insights into the physical processes of earthquake generation. Initially, we divided the data into various time intervals using a sliding window approach. Subsequently, we calculated the *q_M_* values within time intervals containing 1000 events, with a 500-event overlap, resulting in a 50% overlap between consecutive windows. In the statistical analysis we used, in one case, all the seismic events (blue solid line in Figure 4), while in the other case, we focused on events with *M* ≥ *Mc* (red solid line in Figure 4). In the latter case, within each temporal window consisting of 1000 events, we applied the maximum curvature method [35] along with an additional +0.2 correction, to determine the magnitude of completeness (*Mc*). The findings of this analysis are displayed in Figure 4, showing the temporal variations of the *q_M_* values along with their standard deviations. The parameter *q_M_* varies between 1.3 and 1.6 during the studied period. Furthermore, as observed in Figure 4, the *q_M_* value estimation is relatively stable in each temporal window, irrespective of the selection of *Mc*, an important aspect for reliable analysis of the FMD.

The *q_M_* values exhibit an increase during periods characterized by higher-magnitude earthquakes and decrease during more seismically quiet intervals. In Figure 4, we can observe that the largest magnitude events of 1982, 1995, and 2019 induced variations in the values of *q_M_*. The occurrence of such significant events, which are illustrated with stars in Figure 4, causes the increase in the *q_M_* value. We remind that when *q_M_* approaches unity, the system reaches equilibrium and transitions into BG statistical physics. Conversely, as *q_M_* increases, the system deviates from equilibrium and this instability may cause larger magnitude events to occur.

**Figure 5:**
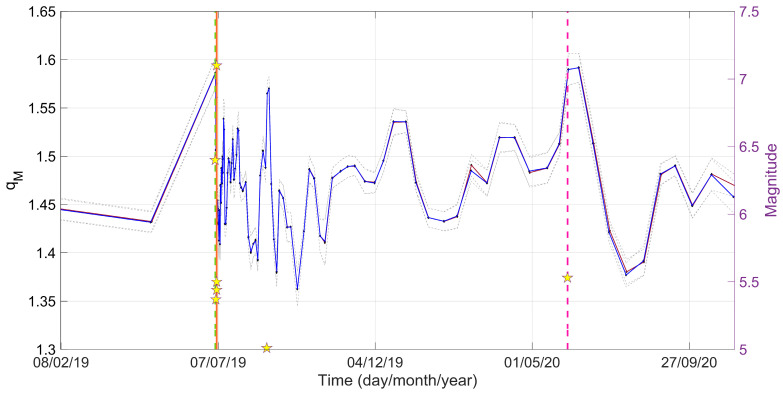
Temporal evolution of the *q_M_* value for all the seismic events (blue solid line) and for *M* ≥ *Mc* (red solid line), along with their corresponding standard deviations (gray dashed lines), calculated in successive time intervals with a 50% overlap. The stars indicate earthquakes of a magnitude greater than 5 during the period from 2019 to 2020. The *M*_w_ 6.4 on 4 July 2019, the *M*_w_ 7.1 on 6 July 2019, and the *M*_w_ 5.53 on 4 June 2020, seismic events are represented with green dashed, orange solid and pink dashed lines, respectively.

In Figure 5, we zoom in into the variations of *q_M_* values from 2019 to 2020, during the period of the Ridgecrest earthquake sequence. The parameter *q_M_* exhibits a notable increase, reaching its peak (*q_M_* = 1.6) on 7 July 2019 and on 4 June 2020, when the *M*_w_ 7.1 and the *M*_w_ 5.53 events occurred. After the strong events, the *q_M_* parameter starts decreasing rapidly.

**Figure 6:**
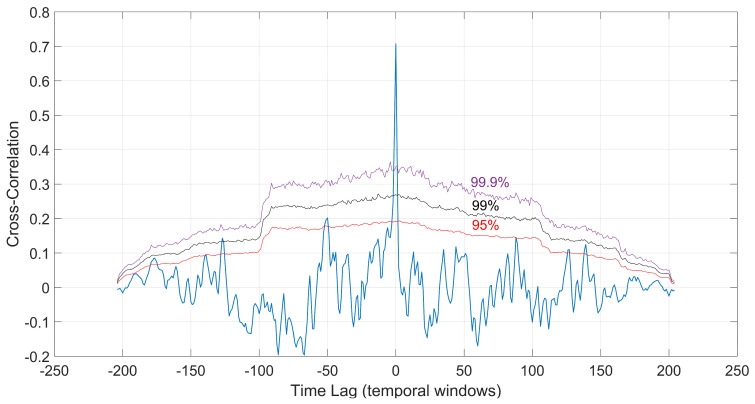
The cross-correlation between the *q_M_* values and cumulative seismic energy *Mo* for the period 1981–2022 and the corresponding 95%, 99%, and 99.9% confidence curves (are labeled with text).

To better resolve the correlations between the released seismic energy and *q_M_* value variations, we perform a cross-correlation analysis [36] designed to quantify the statistical confidence between two datasets, the *q_M_* value and the seismic moment release (*Mo*). This analysis is used to delineate the strength of correlations and the time lag between *q_M_* and *Mo* for the period 1981–2022. Cross-correlation analysis is based on two discrete datasets in time, which are then normalized by subtracting the mean value so that the estimated correlation coefficients range between −1 and 1 [36,37]. The normalized cross-correlation close to zero suggests no correlation, while maximum positive or negative peaks may indicate correlated or anti-correlated signals, respectively. The next step is the use of surrogate reshuffling tests which allow dynamic testing against statistical confidence intervals of anticipated spurious correlations [36]. These tests determine the confidence curves of the estimated normalized cross-correlation. In particular, surrogate datasets of the original series are determined using Monte Carlo methods [36,37]. The reshuffling procedure removes any correlation from the original time series while maintaining their spectral amplitudes in order to enforce the same cyclic autocorrelation [38]. In this test, 10,000 surrogates were computed, and the main peak was observed at cross-correlations greater than 99.9% confidence curves. The cross-correlation between *q_M_* and *Mo* for the time period 1981–2022 is shown in Figure 6 along with the 95%, 99%, and 99.9% confidence curves. We observe nine positive peaks exceeding the 95% confidence curve. The highest peak, with an amplitude of 0.71, occurs with a statistical significance of 99.9% of being non-coincidental. The results of the analysis indicate that the surrogate tests place confidence greater than 99.9% which shows that the *q_M_* value in each temporal window and the corresponding cumulative seismic energy *Mo* are positively correlated.

### 4.3. Spatiotemporal Distributions of q_M_ Values

To investigate the spatiotemporal distributions of *q_M_* values within the activated fault zone based on the F–A model, we proceeded to assess the spatial distribution of the parameter *q_M_* for different time windows. According to the model, the observation of *q_M_* values in space reflects the scale of interactions between fault planes and the fragments that occupy the space between them. Furthermore, an increase in *q_M_* signifies that the physical state moves away from equilibrium in a statistical physics context.

**Figure 7:**
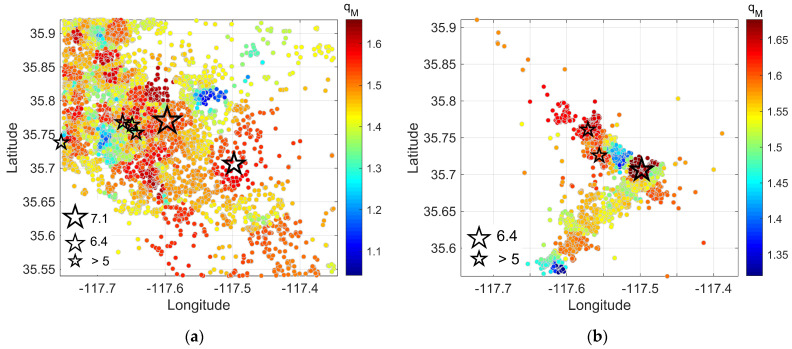
Spatial distribution of *q_M_* values along the 2019 Ridgecrest earthquake fault zone in various time windows, within the studied region defined by coordinates 117.35° E~117.75° E, 35.54° N~35.92° N. (**a**) During the period 1981–2019 (before the *M*_w_ 6.4 foreshock), (**b**) from the *M*_w_ 6.4 foreshock to the *M*_w_ 7.1 mainshock. Larger stars represent the seismic events of *M*_w_ 6.4 and *M*_w_ 7.1, while other stars indicate earthquakes with magnitudes greater than 5, respectively.

Initially, we divided the dataset into temporal segments. The first one covers the period from 1981 to 2019, before the foreshock of magnitude *M*_w_ 6.4 on 4 July 2019 (Figure 7a), while the second, covers the period starting from the foreshock of *M*_w_ 6.4 to the mainshock of *M*_w_ 7.1 on 6 July 2019 (Figure 7b). To estimate the spatial *q_M_* values, we employed the nearest neighbor search method, where for each seismic event, the 200 nearest neighboring events within the dataset were identified. Then, we applied the F–A model to calculate the parameter *q_M_*, using Equation (6). Since *q_M_* remains relatively stable regardless of the choice of completeness magnitude, as it was previously discussed, we opted not to incorporate *Mc* in our calculations. By executing this analysis for each seismic event, we obtained a detailed and comprehensive overview of the *q_M_* parameter in the studied area.

An inspection of Figure 7 indicates that the *q_M_* value ranges from 1.1 to 1.7, supporting subadditivity. In Figure 7a, we observe that the seismicity background is characterized by relatively high *q_M_* values in the areas where the *M*_w_ 6.4 foreshock and the *M*_w_ 7.1 mainshock took place. In Figure 7b, which covers the period from the *M*_w_ 6.4 foreshock to the *M*_w_ 7.1 mainshock on 6 July 2019, we can observe the high *q_M_* values in the area where the *M*_w_ 6.4 occurred. Furthermore, we can observe that the *M*_w_ 7.1 mainshock, located to the NW of the *M*_w_ 6.4 event, occurred in a high *q_M_* value region (Figure 7b).

Moreover, we analyzed the aftershock sequence of the *M*_w_ 7.1 Ridgecrest mainshock from July to November 2019 based on the F–A model. We divided the dataset into four monthly segments, covering the periods from 6 July 2019 (including the *M*_w_ 7.1 mainshock) to 6 August 2019, from 7 August 2019 to 7 September 2019, from 8 September 2019 to 8 October 2019, and from 9 October 2019 to 9 November 2019.

**Figure 8:**
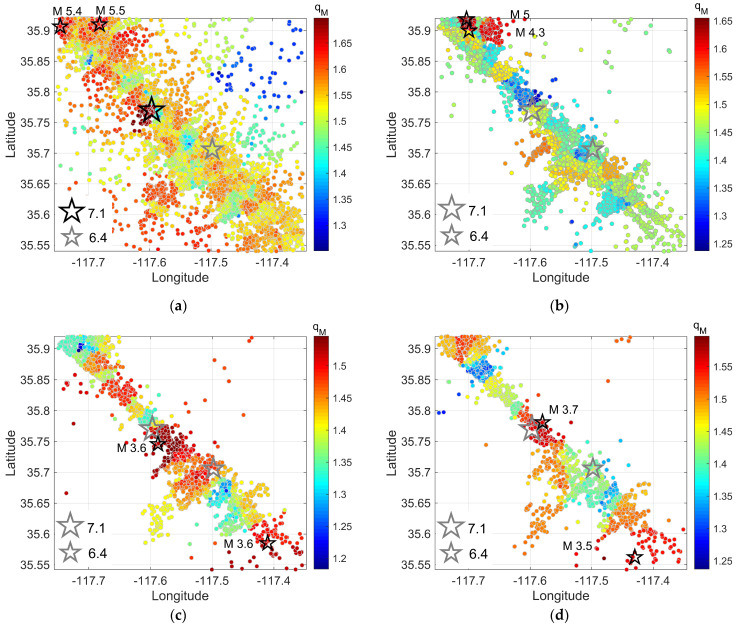
The *q_M_* values along the 2019 activated fault zone. (**a**) From 6 July to 6 August 2019, in the area 117.35° E~117.75° E, 35.54° N~35.92° N. (**b**) The same as (**a**), but for the period from 7 August to 7 September 2019, (**c**) from 8 September to 8 October 2019, (**d**) from 9 October to 9 November 2019. Black stars indicate the strongest events within each month. The gray stars illustrate the locations of the *M*_w_ 6.4 and *M*_w_ 7.1 events.

Two-dimensional spatial analysis (Figure 8) shows that the parameter *q_M_* varies from 1.2 to 1.7 and is high at the locations where the strongest earthquakes occurred. In particular, the black star in Figure 8a indicates the seismic event of *M*_w_ 7.1, with the *q_M_* value reaching as high as 1.7, the highest value in the area. The presence of numerous substantial events with a magnitude of *M* > 4 results in an increase in *q_M_* in the studied area, in which we have depicted the two largest ones to the north of the *M*_w_ 7.1 event (Figure 8a). However, in the same area where the *M*_w_ 7.1 event occurred (Figure 8b), a decrease from 1.7 to 1.45 was observed after 1 month, suggesting stress relaxation in the area of the mainshock. Moreover, in Figure 8b, we observe that the two largest earthquakes during August–September 2019 coincide with the high *q_M_* value regions to the north of the active fault zone. Similar observations are made in Figure 8c,d, where the highest magnitude aftershocks (black stars) coincide with high *q_M_* value areas, particularly in the south and central parts of the activated zone.

## 5. Discussion

In the present work, we used the F–A model, developed within the framework of NESP, to study the temporal and spatial variations of the *q_M_* parameter over the period from 1981 to 2022 in the area of Ridgecrest. The remarkable consistency observed between the F–A model (Equation (6)) and the earthquake magnitude distributions highlights the effectiveness of the F–A model. The parameter *q_M_* informs about the scale of interactions between the fault planes and the fragments that occupy the space between them. When *q_M_* is low (≈1), it indicates the presence of short-ranged spatial correlations and physical states that are near equilibrium [26,39,40,41]. As *q_M_* increases, it signifies a departure from equilibrium in the physical state, suggesting a non-equilibrium state where more earthquakes occur [26]. Regarding the Ridgecrest seismicity during the period 1981–2022, the values obtained for *q_M_* with time vary between 1.3 and 1.6. The observed increase in the entropic index *q_M_* implies that the system is moving further away from an equilibrium state and is in a preparatory process for seismic energy release. In our study, an increase in the *q_M_* parameter can be observed when the major earthquakes of 1982, 1995, 2019, and 2020 occur, with a significant positive correlation between *q_M_* values and seismic moment release throughout the studied period.

Additional studies into the analysis of *q_M_* variations in various cases and within different seismotectonic settings have previously been conducted. In particular, according to [22], the *q_M_* parameter showed a significant increase on 9 April 1994, signaling the onset of a transitional phase leading up to the 1995 Kobe earthquake. Furthermore, the *q_M_* parameter exhibited variations, with an increase observed in the days prior to the strong earthquake of *M_L_* 5.8 in the L’Aquila area (central Italy) [26]. It should be noticed that in [41], a sharp increase in *q_M_* was observed a few days before the occurrence of the significant *M*_w_ 6.4 event in the southwest segment of the Hellenic Arc. Similar research suggests a possible association between *q_M_* and seismicity patterns [40] in the South Pacific coast of Mexico. Moreover, seismic activity in the Hellenic region from 1976 to 2009 was investigated using the method of NESP along with the G–R relation by [42], which concludes that the *q_M_* parameter can be viewed as a distinctive parameter that characterizes the seismic history of a specific region. Previous studies indicate that the NESP approach seems to be a suitable method for analyzing the spatiotemporal patterns of seismicity, as also demonstrated by [28] for the spatial variability of *q_M_* within the Yellowstone Park volcanic region.

Non-extensivity is incorporated in the F–A model as a fundamental statistical component for deriving a cumulative magnitude distribution, of which the Gutenberg–Richter (G–R) relation can be regarded as a specific case [13]. In addition, it is a widely acknowledged and nearly universally observed phenomenon that the stress alterations induced by significant earthquakes have a substantial impact on seismic activity in surrounding areas [43,44]. As suggested by several case studies, mainshock-induced stress changes are therefore anticipated to consistently influence *b* values [35,45,46,47,48,49]. The Ridgecrest earthquake sequence of *M*_w_ 7.1 in California in July 2019 offered an opportunity for [50] to assess both the temporal and spatial variations of the *b* value and its forecasting skills. Hence, a comparison can be made between the fluctuations in the values of *q_M_* and *b*. According to [50], the *b* values were substantially lower after the *M*_w_ 6.4 event compared to the background *b* value, whereas after the mainshock of *M*_w_ 7.1, the *b* value increased within the first week. A decreasing *b* value inside the seismogenic volume has been observed to correspond with increased effective stress levels before significant shocks [51]. Our results for the temporal analysis show that the parameter *q_M_* is higher when the strong events in 2019 occur, and then it decreases over time, in accordance with the results of [50].

Furthermore, the spatiotemporal patterns of variations in *b* values provide additional insights into the prospective location of forthcoming significant events. According to the findings of [50], the *M*_w_ 7.1 event took place near the area of the steepest *b* value decrease. In our study, we can observe that the *M*_w_ 7.1 event occurs in the area where the parameter *q_M_* is increased. The spatiotemporal *q_M_* value distributions, based on the F–A model for each month after the mainshock of *M*_w_ 7.1 can further be compared with the study by [52], in which the aftershock sequence is investigated in terms of the spatiotemporal *b* value distributions within the three-dimensional fault zone. The findings indicate that *b* values were initially homogeneous throughout the spatial area, with a low level of *b* value immediately following the mainshock. However, within 3 months, a rapid increase occurred, reaching a level that is considered typical for California during the interseismic period. As for the parameter *q_M_*, when the *M*_w_ 7.1 occurred, a high value of 1.7 was found in the epicentral area, which is indicated by a black star (Figure 8a). The next month, as we can see in Figure 8b, there was a decrease in the same region (gray star) from 1.7 to 1.45, while an increase in the *q_M_* parameter was observed in the areas where strong aftershocks occurred. Furthermore, the same pattern is observed in the case of the *M*_w_ 6.4 foreshock, where the parameter *q_M_* decreased from 1.7 (Figure 7b) to 1.4 (Figure 8b) within 2 months.

## 6. Conclusions

In the present work, the Ridgecrest earthquake sequence is studied using non-extensive statistical physics (NESP) and the fragment–asperity (F–A) model. Within the context of the F–A model, we calculated the non-extensive parameter *q_M_* and its spatiotemporal variations during 1981–2022, an analysis that informs about the physical state of the studied area. To study the temporal variations of *q_M_* values, we used the entire period and a sliding time window method. Notably, the results show a significant increase in the *q_M_* parameter, which coincides with the occurrence of the strongest earthquakes. Furthermore, it seems that *q_M_* fluctuations over time are a valuable indicator of a seismic area’s physical condition, suggesting different dynamic regimes that can decipher the physical mechanisms leading to a significant seismic event. In addition, we analyzed the seismic events for the spatiotemporal *q_M_* value distributions along the activated fault zone during 1981–2019 and for each month separately after the *M*_w_ 7.1 Ridgecrest earthquake. The results show that *q_M_* values exhibit significant increases in areas where the higher-magnitude events occur, and after the mainshock, *q_M_* values decrease over time, highlighting the stress relaxation process in the activated area.

## Data Availability

Data are openly available at the Southern California Earthquake Data (SCEDC) (https://scedc.caltech.edu/eq-catalogs/altcatalogs.html), accessed on 20 June 2023. The relocated earthquake catalogue is based on the methods described in [31] (https://scedc.caltech.edu/data/alt-2011-dd-hauksson-yang-shearer.html)—last accessed on 20 June 2023.
